# Contribution of Temporal Fine Structure Cues to Concurrent Vowel Identification and Perception of Zebra Speech

**DOI:** 10.1055/s-0044-1785456

**Published:** 2024-07-05

**Authors:** Delora Samantha Serrao, Nikhitha Theruvan, Hasna Fathima, Arivudai Nambi Pitchaimuthu

**Affiliations:** 1 National Hearing Care, Armadale, Australia; 2 Department of Audiology, La Trobe University, Melbourne, Australia; 3 Department of Audiology and Speech-Language Pathology, Kasturba Medical College, Mangalore, Manipal Academy of Higher Education, Manipal, Karnataka, India; 4 Department of Audiology and Speech Language Pathology, National Institute of Speech and Hearing, Trivandrum, Kerala, India; 5 Department of Audiology, Centre for Hearing Science, All India Institute of Speech & Hearing, Mysuru, India

**Keywords:** cochlear implant, speech perception, hearing loss, psychoacoustics, auditory processing, algorithm

## Abstract

**Introduction** The limited access to temporal fine structure (TFS) cues is a reason for reduced speech-in-noise recognition in cochlear implant (CI) users. The CI signal processing schemes like electroacoustic stimulation (EAS) and fine structure processing (FSP) encode TFS in the low frequency whereas theoretical strategies such as frequency amplitude modulation encoder (FAME) encode TFS in all the bands.

**Objective** The present study compared the effect of simulated CI signal processing schemes that either encode no TFS, TFS information in all bands, or TFS only in low-frequency bands on concurrent vowel identification (CVI) and Zebra speech perception (ZSP).

**Methods** Temporal fine structure information was systematically manipulated using a 30-band sine-wave (SV) vocoder. The TFS was either absent (SV) or presented in all the bands as frequency modulations simulating the FAME algorithm or only in bands below 525 Hz to simulate EAS. Concurrent vowel identification and ZSP were measured under each condition in 15 adults with normal hearing.

**Results** The CVI scores did not differ between the 3 schemes (F ^(2, 28)^ = 0.62, *p* = 0.55, η ^2^_p_ = 0.04). The effect of encoding TFS was observed for ZSP (F ^(2, 28)^ = 5.73, *p* = 0.008, η ^2^_p_ = 0.29). Perception of Zebra speech was significantly better with EAS and FAME than with SV. There was no significant difference in ZSP scores obtained with EAS and FAME ( *p* = 1.00)

**Conclusion** For ZSP, the TFS cues from FAME and EAS resulted in equivalent improvements in performance compared to the SV scheme. The presence or absence of TFS did not affect the CVI scores.

## Introduction

The dichotomy of temporal fine structure (TFS) and envelope (ENV) 1 in auditory perception has given a different direction to cochlear implant (CI) research in the last two decades. The conventional CI signal processing discards TFS and preserves only the ENV. The ENV information in a few frequency bands is sufficient for vocoded speech recognition in quiet. 2 Increasing the number of bands containing only the ENV information results in improved speech recognition in quiet but not in the presence of noise. 1 3 4 Lack of TFS is viewed as one of the reasons for poor speech recognition with CI in the presence of noise. 5 6 7 The growing evidence of the role of TFS in the segregation of target speech from the interfering noise, 8 9 10 and dip listening in the presence of fluctuating noise, 11 12 13 14 15 has led to advancements in strategies for encoding TFS in CI. These strategies encode TFS either in the low-frequency bands alone (e.g., electroacoustic stimulation (EAS), fine structure processing (FSP), or on all the available bands (e.g., frequency amplitude modulation encoder (FAME). In EAS, the input signal is low-pass filtered, amplified, and delivered acoustically to the CI users. Individuals with good residual hearing in low frequency can access the TFS cues using EAS. 16 Fine structure processing delivers TFS below 1,000 Hz in the CI as short groups of pulses triggered by negative-to-positive zero crossings in the outputs for selected channels. 17 Frequency amplitude modulation encoder is a theoretical strategy that encodes TFS in CI as slowly varying frequency modulations across all the bands. 9

While the benefit of adding TFS in the low frequencies has been determined, 18 19 it is reported that TFS information over a wide frequency range is essential for perception. 20 Based on the study by Swaminathan and Heinz, 21 it is also clear that TFS in each band contributes to speech recognition in noise as it helps to recover the ENV in each band. However, it is not known if the addition of TFS information to all the bands is advantageous over providing these cues only to the lower bands. Hence, it is essential to compare the performance of strategies that code TFS only in low-frequency bands with a strategy that codes TFS in all the bands.

Stream segregation is one of the mechanisms underlying the role of TFS in speech recognition in the presence of competing speech signals. 9 22 23 The primary advantage of encoding TFS is the facilitation of segregation by effectively transmitting F0 and harmonics. 24 25 Therefore, the performance of EAS and FAME was compared on pitch perception-based stream segregation tasks in the current study. The concurrent vowel identification and Zebra speech perception tasks were used to assess simultaneous 26 and sequential segregation. 27 Behavioral data and model predictions have indicated the predominant role of F0 and harmonics in concurrent vowel perception, 28 29 which justifies the choice of concurrent vowel identification for studying the effect of TFS on concurrent streaming. The concurrent vowel identification paradigm used in the current study was like that 26 in which listeners were required to identify the target vowel in the presence of another competing vowel. Kumar et al. 26 reported a good correlation between concurrent vowel identification ability and speech perception in noise. Zebra speech perception task was chosen for measuring sequential segregation over other measures as it overcomes the effect of regularity on streaming, 30 31 and speech stimuli offer generalization with the real-life speech-in-noise scenario. For both concurrent vowel identification and Zebra speech perception task, the sine-wave vocoder (SV) with no TFS served as the baseline against which the efficacy of EAS and FAME was tested.

## Methods

### Participants

Fifteen native Kannada-speaking adults in the age range of 18 to 25 years participated in each experiment. The hearing thresholds of the participants were ≤ 25 dBHL at octave audiometric test frequencies from 250 Hz to 8 kHz. None of the participants had any history of neurological or cognitive deficits. The institutional ethics committee (approval number: 11-13/202) has approved the study. All the experiments were conducted per the Declaration of Helsinki. 32 Informed consent was obtained from all the participants before conducting the study.

### Stimuli and Instrumentation

Standard sentences from Quick SIN-Kannada 33 were used for the Zebra speech task. The vowels /a/, /e/, /i/, /o/, and /u/ synthesized using a Klatt synthesizer were used for concurrent vowel experiments. A creative sound blaster X-Fi USB2 (Creative labs, Singapore) external sound card and Sennheiser HD280Pro circum-aural headphones (Sennheiser, Wedemark, Germany) were used for stimulus delivery. Stimuli for the experiments were presented at 60 dB SPL. All the experiments were conducted in a sound-treated room.

#### Stimuli Synthesis

##### Concurrent Vowels

Five steady-state vowels /a/, /e/, /i/, /o/, /u/ were synthesized at the sampling rate of 44,100 Hz, using Klatt synthesizer. The vowel /a/ was synthesized with an F0 of 220 Hz and served as the competing vowel. The vowels /e/, /i/, /o/, /u/ were synthesized with F0s, 0, 1, 2, and 4 semitones higher than the F0 of the competing vowel, and these vowels were used as the target vowels. There were 16 target vowel stimuli (4 vowels × 4 F0s). Each vowel had a duration of 290 ms, including 20 ms raised cosine onset/offset ramps. Concurrent vowels were created by adding two vowel pairs after scaling them to the same intensity. Concurrent vowels consisted of the competing vowel paired with one of the target vowels. Formant frequencies and bandwidth for each of the vowels are given in Table 1 .

**Table 1:** Formant frequencies of the synthesized vowels used in the study

	/a/	/e/	/i/	/o/	/u/
F1	840	457	362	480	439
F2	1,309	1,893	1,920	882	814
F3	2,119	2,232	2,265	1,647	1,894
F4	2,576	2,607	2,594	3,219	3,387

##### Zebra Speech

Zebra speech 27 was built from two separate sentences of equal overall intensity of which one sentence served as the target, and the other served as the interferer. A female speaker with an average F0 of approximately equal to 220 Hz produced the target sentence. In the study by Gaudrain and Carlyon, 27 the target sentence was spoken by a speaker with an average F0 of 232 Hz. The F0 of 220 Hz was adopted in the current study to maintain a similar F0 in both concurrent and sequential segregation tasks. A sentence spoken by a male speaker with an average F0 of 100 Hz was the interfering sentence. The root mean square (RMS) amplitude of both sentences was measured in consecutive 40 ms time windows. In each time window, the sentence with the higher RMS was preserved while the other sentence was discarded. If RMS amplitudes of both the sentences were equal, both sentences were retained in that time window. A 5 ms Hann ramp smoothed all transitions between the target and the interferer.

#### Signal Processing

##### Sine-wave Vocoding (SV) and Frequency Amplitude Modulation Encoding (FAME)

sine-wave vocodings were used to simulate CI speech processing using the FAME algorithm. 9 Input speech signals were bandpass filtered into 30 frequency bands using bandpass filters between 80 and 7,563 Hz. Even though conventional CI's do not have 30 bandpass filters, this study used 30 bands to approximate normal cochlear function and to neutralize ENV recovery cues. From each band, amplitude modulation (AM) and frequency modulation (FM) were extracted in two different pathways. In the first pathway, AM was extracted. For AM extraction, the sub-band signals were subjected to full-wave rectification and low-pass filtering at 400 Hz. Such a high AM cut-off frequency was used, as most speech coding strategies of current CI's use a similar cutoff. In another pathway, the sub-band signals were sent through phase orthogonal demodulation filters to remove the center frequency of the signal and retain only the FM. Then, slowly varying FM was extracted by limiting the FM rate to 400 Hz and the FM bandwidth to 500 Hz, or the filter's bandwidth, whichever was less. The SV (AM-only) stimuli were synthesized by modulating the AM on the sine-wave carrier whose frequency is the sub-bands center frequency. The FAME stimuli (AM + FM) were synthesized by frequency modulating the sine-wave carrier whose frequency is the sub-bands center frequency before subjecting to amplitude modulation. Finally, all modulated sub-band signals were summed up. For a detailed description of FAME refer to Nie et al. 9

##### Simulated Electroacoustic Stimulation (EAS)

The FAME algorithm was modified to simulate the EAS. The input stimuli were low-pass filtered at 525 Hz. The high-frequency portion of the signal, above 525 Hz, was bandpass filtered into 21 contiguous frequency bands between 525 and 7,563 Hz. The ENVs' of the signals were extracted by full-wave rectification and low pass filtering at 400 Hz. The ENVs' were then used to amplitude modulate sine-wave carriers whose frequency was the center frequency of bands. Finally, the low-passed signal and modulated sub-band signals were summed up to simulate EAS.

### Procedures

#### The Concurrent Vowel Identification Through FAME, EAS, and SV

The concurrent vowel identification paradigm used in the current study was like the experiments conducted by Kumar et al. 26 The synthesized steady-state vowels /a/, /e/, /i/, /o/, and /u/ were used for the task. The vowels /e/, /i/, /o/, and /u/ served as the targets and /a/ served as the competing vowel. Concurrent vowel stimuli were created by pairing each target vowel with the competing vowel. Participants were instructed to ignore the competing vowel and to identify the target vowel. Following the stimulus presentation, a response box consisting of the four vowels appeared on the screen. Participants responded by clicking on the appropriate button on the screen. Concurrent vowel identification scores were measured as the function of the F0 difference between the target and the competing vowel. Within each concurrent vowel pair, the target and the competing vowel differed in F0 by 0, 1, 2, and 4 semitones. Each concurrent vowel pair was presented 10 times, and the total correct scores were calculated separately for the F0 differences of 0, 1, 2, and 4 semitone conditions. The probability that a vowel was perceived correctly was considered as the hit rate, and the probability of identifying a vowel when other vowels were presented was the false alarm rate. Later d-prime analysis was carried out and d-values were computed for /e/, /i/, /o/ and /u/ for the F0 difference of 0, 1, 2 and 4 semitones.

The experiment was carried out in three conditions. In the first condition, the vowels were processed through EAS. In the second condition, the vowels were processed through FAME. Both these conditions provided TFS information to the participant. In the third condition, no TFS information was given. The order of the presentation of the conditions was randomized.

#### Zebra Speech Through FAME, EAS, and SV

Six lists of standard sentences from Quick SIN-Kannada each containing seven sentences were used to create Zebra speech. These sentences had five keywords in each. The participants were informed that they would be presented with a target sentence spoken by a female speaker along with a competing sentence speaker spoken by a male. They were instructed to attend to the sentence spoken by the female speaker. The participants were asked to write down the words identified. Each correctly identified word was scored 1. The total scores were computed based on the number of correctly identified keywords. The experiment was carried out in three conditions. In one, the sentences were processed through EAS. In the second, the sentences were processed through FAME. Both these conditions provided TFS information to the participant. In the third, the sentences were processed such that no TFS was available to the participant. The order of the presentation of the conditions was randomized.

## Results

### Concurrent Vowel Identification through SV, EAS, and FAME

Two vowels were concurrently presented where the F0s of both vowels differed by 0, 1, 2, and 4 semitones. Vowel pairs were processed through EAS, FAME, and SV algorithms. Each vowel (/e/, /i/, /o/, /u/) was paired with the vowel /a/ and presented 10 times each. The probability of correctly identifying the target vowel was considered as the hit rate. The probability of the target vowel being identified when the other vowels were presented was considered a false alarm. 26 34 The ‘hit’ and ‘false alarm rates’ were further subjected to d-prime analysis. With this method, the maximum possible d-prime score in the current study was 6.18. One sample t-test was administered to investigate whether the d-primes were significantly higher than 0. Table 2 represents the ‘t’ values of one sample t-test for each stimulus condition.

**Table 2:** T-values obtained from the one-sample t-test for the d-prime scores of the identification of concurrent vowels processed through EAS, FAME, and sine-wave vocoder

Processing scheme	Semitone difference	/e/		/i/		/o/		/u/	
		t	*p*	t	*p*	t	*p*	t	*p*
**EAS**	0	3.87*	0.002	4.22*	0.001	5.02*	0.000	4.25*	0.002
	1	3.63*	0.003	3.75*	0.002	2.60	0.021	0.56	0.59
	2	5.65*	0.000	5.24*	0.000	4.34*	0.000	2.45	0.028
	4	4.65*	0.000	5.10*	0.000	5.36*	0.000	5.61*	0.000
**FAME**	0	3.35*	0.005	4.44*	0.001	4.24*	0.001	4.07*	0.001
	1	3.99*	0.001	5.36*	0.000	3.38*	0.005	4.73*	0.000
	2	5.37*	0.000	5.27*	0.000	5.12*	0.000	4.03*	0.001
	4	4.76*	0.000	4.83*	0.000	5.72*	0.000	6.40*	0.00
**SINE-WAVE VOCODER**	0	4.37*	0.001	3.98*	0.001	6.61*	0.000	4.17*	0.001
	1	3.43*	0.004	4.06*	0.001	2.41	0.030	1.34	0.200
	2	4.99*	0.000	6.33*	0.000	5.11*	0.000	2.70*	0.017
	4	4.54*	0.000	4.59*	0.000	7.39*	0.000	5.46*	0.000

Abbreviations: EAS, electroacoustic stimulation; FAME, frequency amplitude modulation encoder.

‘*’ indicates that, d-prime score was significantly different from 0. *P* -level of 0.05 was adjusted using Bonferroni-Holm corrections.

**Fig. 1:**
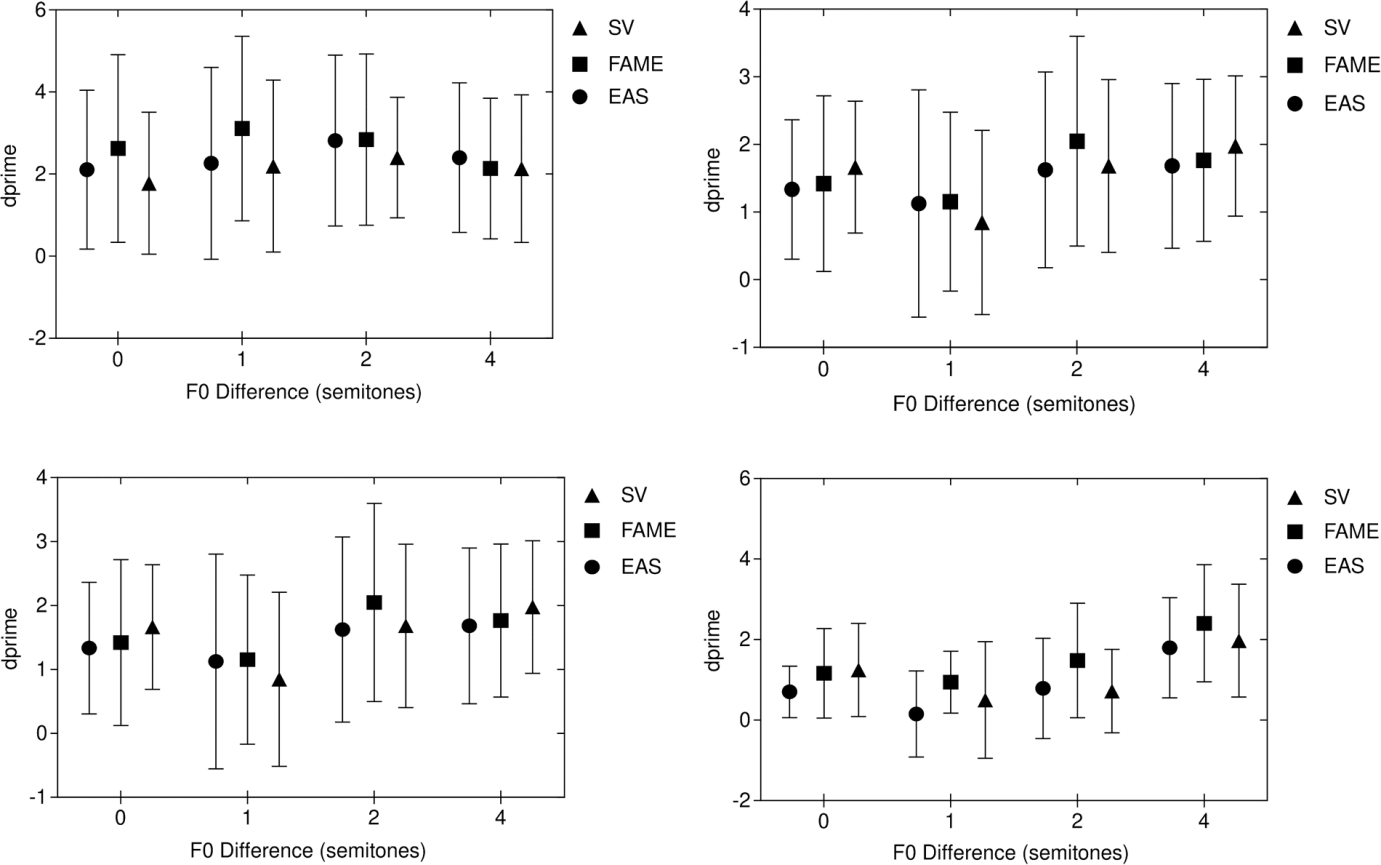
The mean and standard deviation of d-primes for each vowel processed through sine-wave vocoder (SV), Frequency and amplitude modulation encoder (FAME), and electroacoustic stimulation (EAS) (top left: vowel ‘e’, top right: vowel ‘i’, bottom left: vowel ‘o’, bottom right: vowel ‘u’)

A three-way analysis of variance (ANOVA) with repeated measures was performed to investigate the main effect of signal processing schemes, F0 difference (0, 1, 2 & 4 semitones) and vowels (/e/, /i/, /o/ & /u/) on the d-prime score. Analysis revealed a significant main effect of F0 difference (F ^(3, 42)^ = 8.79, *p* < 0.05, η ^2^_p_ = 0.39) and vowels (F ^(3, 42)^ = 8.48, *p* < 0.05, η ^2^_p_ = 0.38) on the d-prime score. Interaction between F0 difference and vowels was also statistically significant (F ^(9, 126)^ = 8.83, *p* < 0.05, η ^2^_p_ = 0.39). An important observation in the study is that there was no significant main effect of the signal processing scheme (F ^(2, 28)^ = 0.74, *p* = 0.49, η ^2^_p_ = 0.05) on d-prime. Also, there was no significant interaction between F0 difference and signal processing scheme (F ^(6, 84)^ = 1.58, *p* = 0.16, η ^2^_p_ = 0.10). The interaction between signal processing scheme and vowels was also not significant (F ^(6, 84)^ = 1.08, *p* = 0.38, η ^2^_p_ = 0.07). These results suggest that participants could identify the concurrent vowels accurately even without the TFS. The mean and standard deviation of d-primes for each vowel processed through the different signal processing schemes are represented in Figure 1 . Since there was a significant interaction between F0 difference and vowels, separate repeated measures of ANOVA were performed to investigate the main effect of F0 difference on the identification of each vowel. For this analysis, the d-prime score for the identification of each vowel at each F0 difference condition was combined across the signal processing schemes. This was done as there was no significant main effect of signal processing, and the signal processing schemes did not have significant interaction with F0 difference and vowels. The F0 difference had a significant main effect on identification of vowel /e/ (F ^(3, 132)^ = 10.58, *p* < 0.05, η ^2^_p_ = 0.19), and the pairwise comparisons with Bonferroni adjustments revealed that d-prime for identification of vowel /e/ was significantly higher for the 2-semitone condition than 0 ( *p* < 0.05), 1 ( *p* < 0.05), and 4-semitone ( *p* < 0.05) conditions. No other comparisons were significant. Similarly, F0 difference had a significant main effect on identification of the vowel /i/ (F ^(3, 132)^ = 3.61, *p* < 0.05, η ^2^_p_ = 0.08). Pairwise comparisons revealed that, identification of the vowel /i/ in the 2-semitone condition was significantly higher than 0 ( *p* < 0.05), 1 ( *p* < 0.05), and ( *p* <0 .05) 4-semitone conditions. Even for the identification of the vowel /o/, the main effect of F0 difference was significant (F ^(3, 132)^ = 7.31, *p* < 0.05, η ^2^_p_ = 0.14). The highest d-prime value was obtained for the 4-semitone difference condition. However, the d-prime for the 4-semitone condition was significantly different only from the 2-semitone difference condition ( *p* < 0.05). The main effect of F0 difference on identification of the vowel /u/ also was significant (F ^(3, 132)^ = 23.35, *p* < 0.05, η2p = 0.35). Like the vowel /o/ and even for the vowel /u/, the d-prime was highest for the 4-semitone difference condition. The pairwise comparisons with Bonferroni adjustments revealed that the d-prime for the 4-semitone was significantly higher than 0 ( *p* < 0.05), 1 ( *p* < 0.05), and the 2-semitone condition ( *p* < 0.05).

### SINFA Analysis

The stimulus-response matrix was constructed based on the responses for the target vowel presentation. Matrices were then subjected to Sequential Information Transfer Function Analysis (SINFA) using the Feature Information Xfer (FIX) software (University College of London, England). The matrices were analyzed for information transmitted and the proportion of correct responses. Sequential Information Transfer Function Analysis is advantageous in cases where the listener's responses are independent of the stimuli presented. For example, chance performance can yield different percent correct scores, depending on how speech stimuli are categorized into features. Also, SINFA offers information about perceptual features ingrained in a stimulus-response matrix and estimates the proportion of the information transfer for a given set of perceptual features. Hence, for the SINFA, stimuli need to be categorized based on perceptual or phonological features. In the present study, the vowels were categorized based on the features of tongue height, lip rounding, and place of articulation. The *place* feature classified the vowels as front or back, lip rounding consisted of rounded or unrounded options, and tongue height classified the vowels as high or low. The chance performance for each vowel identification is 25%. However, if the vowels are categorized based on the lip rounding feature (rounded vowels vs. unrounded vowels), the chance-performance would be 50%. Similarly, chance performance would be 50% when the vowels are classified based on the place and tongue height of the vowel. In these cases, the stimuli-specific percent correct scoring would be anomalous if the participant's responses are biased despite the responses being independent of the stimuli presented. However, the information transfer would be 0%, thus efficiently accounting for the listener's bias. 35 Hence, in the current study, the stimulus-response matrix for the target vowel identification was further subjected to SINFA. After the vowels were categorized into features, the information transfer per feature was calculated by a sequence of iterations in which one feature was partialled out per iteration by holding that feature constant. For a detailed description refer to. 36 The features of the matrices used to categorize vowels are represented in Table 3 .

**Table 3:** Features of the matrices according to which the vowels were classified

	/e/	/i/	/o/	/u/
Place	Back	Back	Front	Front
Lip rounding	Unrounded	Unrounded	Rounded	Rounded
Tongue height	Low	High	Low	High

A three-way ANOVA with repeated measures was done to evaluate the information transfer under different processing schemes. For this analysis, processing schemes (EAS, FAME, and SV speech), F0-difference (0, 1, 2, and 4 semitone difference between the concurrent vowels), and features (place, lip rounding, and tongue height) served as the independent variables. Results revealed that there was no effect of processing scheme on information transfer (F ^(2, 28)^ = 0.62, *p* = 0.55, η ^2^_p_ = 0.04). However, significant effect of F0 difference (F ^(3, 42)^ = 6.18, *p* < 0.05, η ^2^_p_ = 0.31) and features (F ^(2, 14)^ = 39.21, *p* < 0.05, η ^2^_p_ = 0.74) on information transfer was seen. The mean and standard deviation information transmission scores for each feature under the three processing schemes in 0, 1, 2, and 4 semitone conditions are represented in Table 4 .

**Table 4:** Mean and standard deviation of information transfer for the EAS, FAME, and SV speech with 0, 1, 2, and 4 semitone differences

Processing	Semitone difference	Place/Lip rounding		Height	
		Mean	Standard deviation	Mean	Standard deviation
EAS	0	0.54	0.31	0.19	0.15
	1	0.59	0.37	0.34	0.28
	2	0.66	0.33	0.37	0.30
	4	0.68	0.34	0.31	0.23
FAME	0	0.68	0.33	0.32	0.17
	1	0.66	0.33	0.31	0.23
	2	0.72	0.30	0.41	0.23
	4	0.72	0.32	0.28	0.20
Sine-wave vocoder	0	0.67	0.25	0.23	0.24
	1	0.64	0.30	0.26	0.22
	2	0.66	0.28	0.36	0.29
	4	0.69	0.29	0.27	0.23

Abbreviations: EAS, electroacoustic stimulation; FAME, frequency amplitude modulation encoder; SV, sine-wave vocoder.

### Perception of Zebra Speech through SV, EAS, and FAME

The perception of Zebra speech processed through EAS, FAME, and SV condition was assessed. The participants were asked to attend to the target stimuli and repeat the sentence. Scores were based on the number of keywords correctly identified by the participants in the three conditions. The mean and standard deviation of the total correct scores of the participants are represented in Table 5 .

**Table 5:** Mean and standard deviation of total correct scores for Zebra speech processed through EAS, FAME, and SV conditions. The maximum possible score is 35

	**Mean**	**Standard deviation**
EAS	22.53	8.63
FAME	23.93	7.28
SV	17.87	8.62

Abbreviations: EAS, electroacoustic stimulation; FAME, frequency amplitude modulation encoder; SV, sine-wave vocoder.

One-way ANOVA with repeated measures was used to evaluate the effect of processing strategy on the perception of Zebra speech. Before the analysis, total correct scores were converted into rationalized arcsine units (RAUs) scores to account for the critical differences such as floor and/or ceiling effects present in the conventional scoring method (Studebaker, 1985). The following formula proposed by Sherbecoe and Studebaker 37 was used to convert total correct scores into RAU scores.

 (1)

Here, *‘x’* is the score obtained by the participants and *‘n’* is the total possible score.

 (2)

One-way ANOVA with repeated measures revealed that signal processing strategy had a significant main effect on Zebra speech perception (F ^(2, 28)^ = 5.73, *p* = 0.008, η ^2^_p_ = 0.29). The pairwise comparisons with Bonferroni corrections showed that the perception through EAS ( *p* = 0.01) and FAME ( *p* = 0.03) was significantly different from the perception of SV speech. Perception of Zebra speech through EAS and FAME was significantly better than SV speech. There was no significant difference between the perception of stimuli processed through EAS and FAME ( *p* = 1.00).

## Discussion

### Concurrent Vowel Identification

In the concurrent vowel identification task, performance was estimated as a function of the differences in F0 between the vowels and as a function of processing strategies (EAS, FAME, and SV vocoder). Since the TFS effectively carries pitch information, it was predicted that d-prime for concurrent vowel identification would be better in EAS and FAME than in SV. However, the d-prime and SINFA analyses revealed that there was no significant difference between the strategies in terms of concurrent vowel identification ability. Also, there was no significant interaction between processing strategies and the F0 difference. These results suggest that TFS is not essential for the identification of concurrent vowels. Alternatively, it can be interpreted that the F0 differences, which are effectively carried by the TFS, may not be essential for concurrent vowel identification.

In the current study, the identification of the target vowel may be limited by factors including the (i) increase in the internal noise due to the masking effect by the interfering vowel, or (ii) failure of segregation resulting in the perception of both vowels as a single auditory object or two indistinguishable objects. F0 and its harmonics are viewed as the essential cue to overcome the above-mentioned limiting factors 26 as the F0 and harmonics information helps to segregate the target vowel and interfering vowel into separate streams. However, in the current study, d-values were significantly above 0 even for the 0-semitone condition indicating that discrimination occurred even when there were no F0 differences. Mean values did not show a clear trend of increasing d with increasing differences in F0 even through FAME. As discussed above, the F0 advantage may not be required for the concurrent vowel identification at least in the current study's paradigm. In a similar study, improvement in vowel identification was observed for a 1-semitone difference, while it is known that at least a 4-semitone difference is essential for perceiving two pitches as distinct. 38 Computational and behavioral data also support the argument that there might be factors other than the F0 and harmonics contributing to vowel identification 39 40 41 42 and that the F0 benefit might vary with duration and level of stimuli. 43

An important question that arises here is, if not F0, what are the other possible cues that have contributed to the identification performance? For the experimental paradigm used in the current study, the spectral shape would have played a major role as it is an important cue to determine the identity of the vowels. 44 It is expected that spectral shape information is conveyed better through FAME than through EAS and SV. In FAME, before ENV modulation, the sinusoidal carrier is frequency modulated. Hence, spectral bandwidth information is conveyed in FAME, whereas in EAS and SV speech, ENV modulation was done on a single sine-wave carrier. So, it can be expected that identification performance would be better in FAME than in EAS and SV speech. However, there was no significant difference found in d between the three algorithms in the current study, and this can be attributed to the sideband bands created by the ENV modulations. 45 When a high ENV cut-off (like the one used in the current study = 400 Hz) is used, the ENV will carry the information of F0. When this ENV is used to modulate the sine-wave carrier, the sidebands will appear on both sides of the carrier frequency. These sidebands are likely to occur at frequencies equivalent to the sum and difference of the carrier frequency and F0. These sidebands create harmonic-like spectral components at the output of each channel. 45 When the output of all the channels is combined, overall spectral shape information is obtained. While the SV speech lacks spectral information at the input level, some amount of information can be retrieved based on the comparison of amplitude changes across channels over time. 46 Since all three schemes can carry spectral shape information, identification performance can be justified based on spectral shape cues. On the other hand, filtering and modulation introduce slight spectral smearing. 2 This spectral smearing could be the reason why no participants obtained perfect d in any of the processing conditions. Another observation in the study is that F0 differences had a significant main effect on vowel identification. For the identification of the vowels /e/ and /i/, the maximum d-prime was obtained at the 2-semitone difference condition. However, for the identification of the vowels /o/ and /u/, the maximum d-prime was obtained at a 4-semitone difference condition. These results suggest that the effect of the F0 difference on concurrent vowel identification is vowel specific. Similarly, de Cheveigne 28 reported a vowel-specific effect of F0 difference on concurrent vowel identification ability. The results of the current study give a notion that a change in the F0 may influence the spectral shape in some manner which may be beneficial to identifying some vowels at certain F0 difference conditions.

### Zebra Speech

In the present study, performance was found to be significantly better for Zebra speech processed through EAS and FAME when compared to SV speech. The results of the current study suggest that TFS is essential for sequential stream segregation.

Both sentences in the Zebra speech used in the study differed in pitch, and pitch is reported to be an important factor in the segregation of speech from different sources. 47 It is reported that pitch coded by the ENV periodicity does not serve as a prominent cue to segregation, 48 as the pitch information carried by the ENV is weak. Also, Manjunath et al. 49 reported that ENV periodicity corresponding to F0 failed to contribute to concurrent stream segregation, which explains the poor performance of participants in the SV condition. It was also found that the addition of TFS in the form of EAS or FAME contributed significantly to sequential segregation by better coding of F0 and harmonicity. In Zebra speech, the target speech is available to the listener at discrete time segments. Pitch information helps link these across time segments of the target sentence and integrate this information over time, thereby strengthening the sequential segregation process. Temporal fine structure information would have led to better coding of the pitch, thus helping integrate the across-time segments of the sentences having the same pitch into a single stream. If the across-time segments of the sentences have a different pitch, they would be segregated into different streams.

Even though the EAS codes TFS only in low frequencies, the perception of Zebra speech through EAS was not different from FAME. This result suggests that EAS codes the pitch information as well as FAME processing that is necessary for Zebra speech perception. Pitch information is strongly coded through lower resolved harmonics than higher unresolved ones. 50 In the Zebra speech task, the participants were instructed to ignore the interfering speech spoken by the male speaker whose F0 is 100 Hz. In the EAS processing, the input speech was low-pass filtered at 525 Hz. This would have allowed better coding of F0 and the first 4 harmonics of interfering speech, which would have helped the listener estimate the pitch of the interfering speech. Using this pitch information, the listeners would have segregated the interfering speech from the target speech. Also, studies have shown that TFS in low-frequency bands contributes more to speech intelligibility than TFS in high-frequency bands. 20 51 52 Since the EAS processing makes the TFS available in low-frequency bands, it would have produced similar speech recognition scores as those of FAME processing.

## Conclusion

The current study assessed the role of TFS in simultaneous and sequential stream segregation. Temporal fine structure information was either absent, presented to all frequency bands, or limited only to low-frequency bands. Temporal fine structure was presented to all frequency bands using the FAME algorithm and restricted only to low-frequency bands through EAS simulation. Simultaneous and sequential stream segregation was assessed using concurrent vowel identification and Zebra speech task, respectively.

The addition of TFS cues did not improve the concurrent vowel identification ability , thus suggesting that TFS cues may not be essential for concurrent vowel identification. On the other hand, the perception of Zebra speech was significantly improved when TFS cues were added. The improvement was similar when TFS was restricted only to low-frequency bands or was added to all bands. The result indicates the importance of providing TFS information in the low-frequency bands for sequential segregation of speech. The findings of the current study have clinical implications by showing that FSP and EAS strategies, coding TFS in low-frequency bands only, may be sufficient for sequential segregation. Future coding strategies may then focus on transmitting TFS in low-frequency bands only. However, the results of the current study must be cautiously interpreted as the number of analysis and synthesis bands used in the current study is slightly higher than the number of bands used in the clinical speech coding strategies.
